# In this issue of *SNI* — June 2010

**DOI:** 10.4103/2152-7806.65052

**Published:** 2010-07-01

**Authors:** James I Ausman

**Affiliations:** Editor-in-Chief, *Surgical Neurology International*, USA

## *SNI:* JUNE 2010, NEW FEATURES

You will see more changes this month in *SNI*. Look at the Tool Bar; under “Multimedia” see more lectures and “How I do it” videos of surgery. Click “Forum” and see the options for you to add challenging cases under “Difficult Cases” for worldwide opinion. For “Operative Technique” questions, you can ask for ideas or opinions or add “tips” like the discussion of Keen's Point, Kocher's Point, Dandy's Point and Frazier's Point for entry into the ventricle. We have introduced a “News” section, which has news about neurosurgeons, national societies and meetings. We have also added an *SNI* “Worldwide Market” where you can ask for or donate equipment, find fellowships or volunteer. You can comment or add to any of these sites after a simple login that protects *SNI* from unwanted entries. This is easy to do.

## *SNI:* JUNE 2010 PAPERS

I have written a brief summary of some important papers in science and translational neuroscience that our readers should know. The first paper describes a landmark achievement in science in developing a synthetic DNA and transferring it to another bacterial cell to produce the donating strain of bacteria in replication. This is the creation of life in the laboratory in its broadest sense. The second paper reviews the factors, molecular pathways, genetic changes, environment, social forces, and multi-systems involved in addiction to nicotine. This paper is an example of what happens in addiction.

Peter Jannetta and his colleagues have proposed a central nervous system (CNS) basis as a cause of type 2 diabetes mellitus (DM). Based on a previous observational study of 15 patients with cranial nerve dysfunction and with right lateral medullary vascular compression of the brainstem, Jannetta noted that some of these patients had a reduction in their blood sugar levels after surgery. As a result of these clinical observations, the authors preformed a prospective study of patients with type 2 DM who also had right lateral medullary brainstem vascular compression. Patients with significant vascular compression and distortion of the right lateral medulla on magnetic resonance (MR) were selected for surgery. Of the 10 patients in the study, 7 showed significant improvement in their glucose control. Those who did not improve had a significantly higher body mass index. The observations in this study cannot be disputed. Jannetta has also found that hypertension can be relieved in selected patients with left lateral medullary vascular compression. It is becoming well recognized that the CNS is involved in the development of many systemic diseases through the autonomic system. As neurosurgeons, we see cardiac changes with subarachnoid hemorrhage (SAH), and neurogenic pulmonary edema. There is evidence suggesting regional enteritis, psoriasis, rheumatoid arthritis, autoimmune diseases and immunity, gastric ulcers, and other diseases are intimately connected to nervous system function. Exploration of the role of the CNS in these diseases will be a fundamental path of medicine in the 21^st^ century. Thus, the results of this study on right lateral medullary compression as a cause of type 2 DM need to be further explored by others so that we can have a greater understanding of the CNS relationship to this disease. This is an outstanding preliminary study of CNS relationships to type 2 DM and leads the way to other investigations of CNS–systemic disease connections. Note how this concept can be related to the multiple factors and systems involved in drug addiction discussed in the translational neuroscience paper.

Henderson *et al*. have reported a superb study in which the deformative stress on the brainstem as a result of basilar invagination is analyzed. Using finite-element analysis, a physical technique that measures the pixel forces in the brainstem, the authors determined that in a selected group of 5 patients with Chiari I malformation or basilar invagination and associated clinical symptomatology, suboccipital decompression and reversal of the abnormal clival angle with fixation improved both the symptoms, and the finite-element analysis determined stresses on the neural tissue. The study was done with objective details in all areas. This is a fitting paper to follow the Jannetta paper mentioned above, as they both indicate that other extra-parenchymal forces can alter the neural function of structures in the brainstem. Read the ‘Discussion’ of this paper for an excellent review of the problem and the literature. Both the Jannetta and Henderson papers indicate approaches neurosurgeons can take in the future to combine skills and knowledge with other specialties to make major advances in medicine.

Spallone *et al*. describe their technique using a transcranial approach to pituitary tumors invading the cavernous sinus. The paper is valuable for its description of the technique of directly visualizing the carotid, optic, third and fourth nerves to remove tumors that have invaded the cavernous sinus. The advantages of this approach to the subtotal removal that can be achieved through transnasal endoscopic methods are stated. For benign tumors such as these, this approach leads to total removal and can be also utilized in areas of the world where other technology and radiation facilities are not available. This is a thoughtful piece of work.

Alves *et al*. review the literature on the use of topical antibiotics in neurosurgery to prevent infection. As you would expect, there are no randomized studies on this subject, although many neurosurgeons utilize topical antibiotics. The ‘Discussion’ of the paper is worth reading as it provides a knowledge basis for what many of us do in surgery today. Especially interesting are the historical citations that explain why the topical agents were used initially. The Malis approach to produce 0% infections is detailed. It is time for a thoughtful randomized trial to decide what antibiotic prophylaxis is necessary for surgery.

Ghaly *et al*. describe a case of perioperative embolic cerebrovascular infarction in a 53-year-old male undergoing a radical prostatectomy under anesthesia. Ghaly, who is also a Board-certified anesthesiologist, suggests the establishment of definite criteria for neurological assessment in the perioperative period so that appropriate treatment, such as clot removal by medicine, endovascular techniques or surgery, can be achieved if indicated.

Pieter Kubben, the *SNI* Neurosurgery 2.0 Editor, has written a very simple explanation for everyone on what “Wiki,” “YouTube,” “Podcasts,” “RSS Feeds,” “Blogs,” “Twitter” and other social networks mean to *SNI* readers. *SNI* has incorporated many of these new Web 2.0 applications in its format so that our readers of all ages can have access to *SNI* information, which is becoming very diverse and which utilizes Web 2.0 (Neurosurgery 2.0) applications. His editorial is the best I have read to explain in a brief way the changing world of the Internet in which we live. This is a ‘must read.’

In the Guest Editorial for June 2010, Morris Beschloss, a nationally recognized financial analyst and newspaper columnist, has written what he sees as the forces shaping the economic future of the USA and other countries in the world.

Pat Kelly has written an editorial on “Liberty and Injustice for All,” a comment on the developing malpractice climates in many countries.

Look for the papers I did not mention, in the June 2010 issue.

